# Internet-Based Prevention Program of Victimization for Youth in Care and Care Leavers (EMPOWER YOUTH): Protocol for a Randomized Controlled Trial

**DOI:** 10.2196/34706

**Published:** 2022-06-14

**Authors:** Birgit Wagner, Laurence Reuter, Betteke Maria van Noort

**Affiliations:** 1 Department of Psychology Medical School Berlin Berlin Germany

**Keywords:** foster care, youth in care, victimization, prevention, online program

## Abstract

**Background:**

The global estimate of the number of children in institutional care is around 5 million, with around 1 million of these children living in Europe. In Germany, about 75,000 children and adolescents find themselves in the foster care system and about 93,000 additional children and adolescents are living in institutions. Traumatic experiences and neglect in childhood are highly prevalent among these youth in care and are related to severe long-term effects. Childhood maltreatment and abuse can increase the risk of future victimization experiences. Although youth in care are at risk of victimization or revictimization, no specific evidence-based prevention program has been designed to address these specific needs.

**Objective:**

This study aims to evaluate the efficacy of a newly developed 6-module internet-based prevention program of victimization for youth in care, named EMPOWER YOUTH.

**Methods:**

In a randomized controlled trial, the intervention group will be compared to a waiting-list control group with an unblinded 1:1 allocation ratio. Assessments will take place before randomization (baseline) and at follow-up 18 weeks after baseline (ie, 12 weeks after finishing the last module of the program). The primary endpoint is the number of victimization, and online and offline bullying experiences (composite score) at the 18-week follow-up. Secondary endpoints are risk-taking behavior, aggressive tendencies, empathy, prosocial behavior, depressiveness, and loneliness at follow-up. The expected outcome requires a sample size of 156 subjects to achieve a power of 80%. Assuming a 30% dropout rate at follow-up, we require 225 participants to be allocated to the trial. Participants are youth in care, that is, adolescents in foster care, adopted adolescents, or young care leavers aged 14 to 21 years.

**Results:**

Ethical approval was granted by the Ethics Committee of the Medical School Berlin in March 2021 (MSB-2021/55). Recruitment started in September 2021 and is planned until November 2022. The results are expected to be published in January 2023.

**Conclusions:**

Given the increased likelihood for future victimization experiences among youth in care, there is a strong need for a low-threshold intervention specifically for this high-risk age group. There are no existing nationwide mental health programs exclusively for youth in care in Germany.

**Trial Registration:**

German Clinical Trials Register DRKS00024749; https://tinyurl.com/tjaahayw

**International Registered Report Identifier (IRRID):**

DERR1-10.2196/34706

## Introduction

### Background

The global estimate of the number of children in institutional care is around 5 million, with around 1 million of these children living in Europe [1]. Reliable data on the number of children living in foster care or adoptive families worldwide are currently not available. In Germany, about 75,000 children and adolescents find themselves in the foster care system and about 93,000 additional children and adolescents are living in institutions [2]. Traumatic experiences and neglect in childhood are highly prevalent among these youth in care and are related to severe long-term effects on mental health [3]. Childhood abuse and neglect are associated with high levels of symptoms such as sexualized behavior, anxiety, posttraumatic stress disorder (PTSD), and depression [4]. Furthermore, an abusive or neglected caretaking setting can be a risk factor for continued and repeated negative relationships. Often, the interpersonal and intrapersonal beliefs of abused individuals are strongly negative about themselves and others, and low self-esteem in youth in care has been found in a number of studies [5]. Moreover, youth in care often show insecure and disorganized attachment behavior [6] and have long-term interpersonal difficulties, disturbances of self, and impaired affect regulation.

Consequences can be multifaceted. Youth in care have a greater risk of unintended pregnancy, ranging from 16% to 50% [7]. Moreover, risky sexual behaviors, including initiating sexual intercourse at an earlier age, having a greater number of sexual partners, inconsistently using contraception, and exchanging sex for money, have been found in this group of adolescents [7]. Stevens et al in their study with youth in care found that higher levels of anxiety and depression were related to higher rates of risky sexual activity and substance use [8]. Often, the ability of risk recognition, which is the ability to identify danger cues (eg, dangerous social situations), is decreased [9]. These factors can lead to interpersonal high-risk situations, which may hinder proper responses and may lead to future victimization. Childhood maltreatment and sexual abuse are associated with a 2 to 3 times higher risk of revictimization [10]. In conclusion, youth in care comprise a highly vulnerable group in adolescence and young adulthood. They often demonstrate poor risk recognition and are often victims of bullying (online and face-to-face), sexual assault, and maltreatment. A change in the caretaking setting via an out-of-home placement in either a foster care family [3,11] or an institution [12] does not prevent this increased chance for future maltreatment or the long-term negative mental health outcomes.

Although the majority of youth in care are at high risk of victimization or revictimization, no specific evidence-based prevention program has been designed to address these specific needs. Most interventions aimed at youth in care are multidimensional programs of a heterogeneous nature that offer a broad focus of treatment (eg, cognitive behavioral techniques, psychoeducation, case management, skill building, emotional literacy, and social support). A systematic review by Hambrick et al evaluated mental health interventions for children in foster care (aged 0-12 years) [13]. Medium effect sizes were found for decreased internalizing problems, and large effect sizes could be shown for positive parenting practices. However, only 3 of the studies were randomized controlled trials (RCTs) [13]. In a more recent systematic review, Bergström et al concluded that only 3 of 18 included interventions for youth in care had sufficient support for program efficacy [14] (Attachment and Biobehavioral Catch-Up [15], Incredible Years [16], and Take Charge [17]). The reported effect sizes were small to moderate [14]. However, none of the included programs focused on the prevention of future victimization [13,14]. Moreover, most programs target foster parents or social workers and not youth in care directly, and take place face-to-face, often in group settings, creating several challenges for feasible widespread implementation of these programs.

Only few studies have been conducted for the prevention of sexual revictimization of adolescents. DePrince et al evaluated prevention of revictimization of adolescent girls in the child welfare system [18]. The participants in the risk detection group were about 5 times less likely to report sexual revictimization compared to the nonintervention control group. A group prevention program for sexual revictimization using risk recognition, communication skills, practical knowledge teaching (ie, not leaving the party with a stranger and refusing alcohol), and problem-solving strategies could reduce the incidence of sexual assault [19]. Further, the program significantly increased self-efficacy and decreased distress at follow-up. Another revictimization program for women with a history of childhood sexual assault was based on acceptance and mindfulness-based theory but failed to find significant differences between the intervention and control groups for revictimization [20]. A computer-based program aimed at preventing dating violence and sexual victimization was examined in schools with youth aged 11 to 15 years [21]. The Me & You computer-based program was found to significantly lower the odds for perpetrating dating violence, but not to lower the odds for victimization [21].

In the past years, computer-based interventions have been developed through the integration of technology and psychological interventions. Internet-delivered interventions have a number of advantages compared to traditional face-to-face treatments for adolescents and young adults. First, this age group has a high affinity for the use of the internet, online games, and social networks. Further, face-to-face interventions are often restricted by long waiting lists, low availability of psychosocial support, and time constraints. Finally, the anonymity of the internet offers participants an alternative way to overcome their initial shame and encourages them to confront difficult themes, such as social difficulties, and to disclose feelings of shame [22]. An increasing number of e-mental health studies have been conducted in youth for a variety of psychiatric and somatic conditions. A meta-analysis [23] found an overall pooled effect size with *d*=0.85 for internet-delivered cognitive behavioral therapy in youth, with a large effect for psychiatric conditions (*d*=1.27) and a lower treatment effect for somatic conditions (*d*=0.49). Systematic reviews and meta-analyses showed that cognitive behavioral web-based interventions for individuals with PTSD symptoms [24] and youth with neurodevelopmental disorders, anxiety, depression, and even suicidal ideation had high efficacy [25-27] comparable to traditional face-to-face settings [28,29].

In summary, youth in care are in clear need of additional support to prevent future victimization. Considering the age group, a guided web-based low-threshold program is a more novel and preferred mode of delivery, given the ubiquitous digital activity in this group. An internet-based intervention has several advantages compared to traditional prevention programs conducted in a face-to-face setting [29]. So far, no program specifically targeting the prevention of several forms of victimization in youth in care has been developed, and no clinical trial is currently being conducted for examining an internet-based intervention to prevent victimization or revictimization among youth in care.

### Objectives and Trial Design

We aim to evaluate the efficacy of a newly developed 6-module internet-based prevention program of victimization among youth in care, named EMPOWER YOUTH. Intervention effects will be evaluated within an RCT (German Clinical Trials Register DRKS00024749) comparing the program with a waiting control group, with an unblinded 1:1 allocation ratio. At initial assessment, participants will be blinded to their allocation status. Assessments will take place before randomization (baseline) and at a follow-up 18 weeks after baseline (ie, 12 weeks after finishing the last module of the program). The main goal of the program is to analyze the spillover effects of the prevention coaching program EMPOWER YOUTH on victimization. The primary endpoint is the number of victimization, and online and offline bullying (composite score) experiences at the 18-week follow-up. Secondary endpoints are risk-taking behavior, aggressive tendencies, empathy, prosocial behavior, depressiveness, and loneliness at follow-up. We hypothesize that the prevention program will be effective for reducing the incidence of victimization, improving coping mechanisms in problematic social situations, and increasing the recognition of risk/dangerous situations.

## Methods

### Research Consortium

This project is supported by a research grant (grant number FKZ 01KR1806E) of the Federal Ministry of Education and Research in Germany and is part of a research consortium called EMPOWERYOU, which is made up of the following universities and institutions (in alphabetical order): Karlsruhe Institute of Technology, Medical School Berlin, PFAD e.V. Association of Foster Care and Adoptive Families in Germany, University of Aachen, University of Bielefeld, and University of Bremen. The study protocol follows the SPIRIT (Standard Protocol Items: Recommendations for Interventional Trials) guidelines for reporting clinical trial protocols [30].

### Recruitment

Youth in care are defined as adolescents in foster care, adopted adolescents, or young care leavers aged 14 to 21 years. Participants will be recruited through the organization PFAD e.V. Association of Foster Care and Adoptive Families in Germany, as well as other organizations working with youth in care through which more than 13,000 foster families or young care leavers can be reached. Since many youth in care are not organized in groups or organizations, recruitment will also take place online via social media (ie, Instagram, Facebook, and TikTok). Furthermore, flyers and posters will be sent to child welfare services, as well as other welfare institutions working with youth in care in Germany.

### Participants and Eligibility Criteria

#### Inclusion Criteria

**Figure 1:**
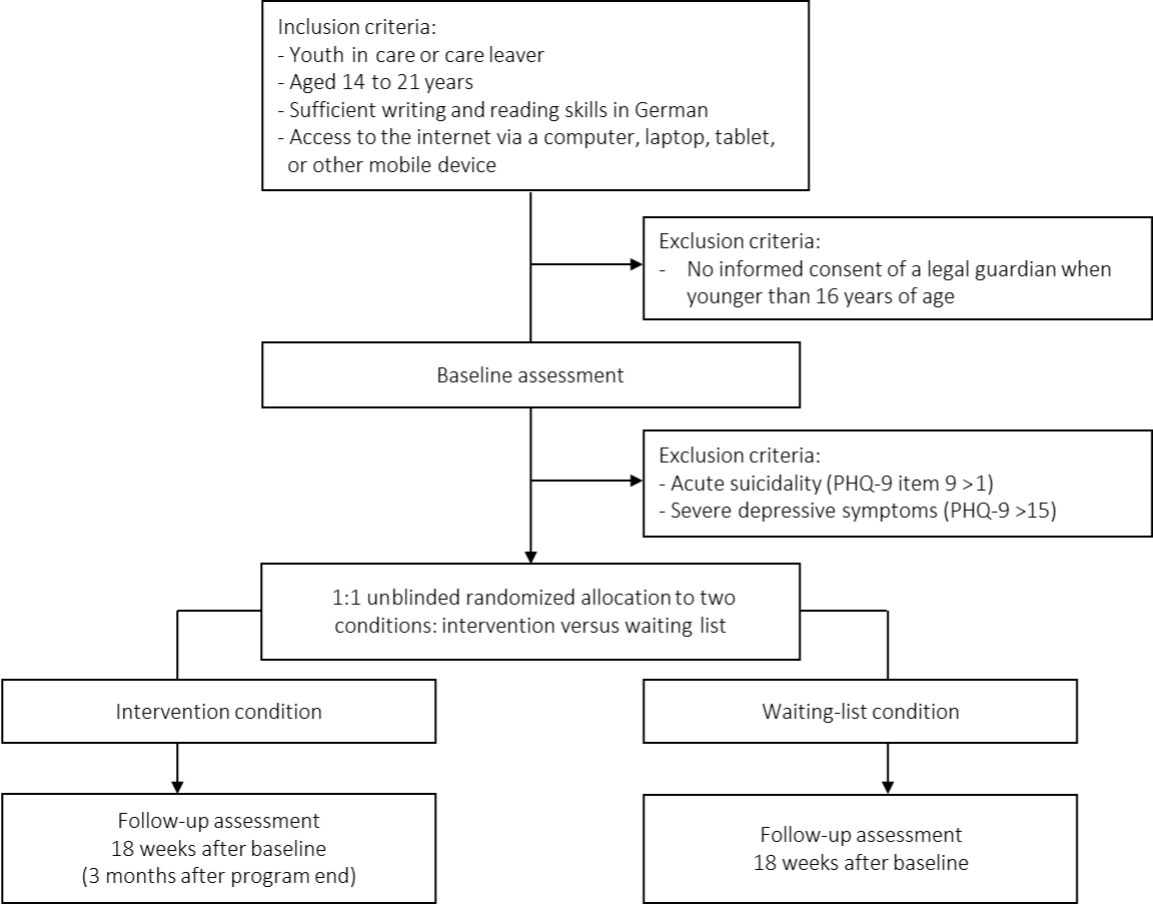
Flowchart of the EMPOWER YOUTH randomized controlled trial.

The study flowchart is provided in Figure 1. Youth in care are defined as adolescents in foster care, adopted adolescents, or young care leavers aged 14 to 21 years. The age range 14 to 21 years is consistent with the definition for “adolescents” in the German Guidelines for Psychotherapy [31] (article A, first paragraph, point 4). Moreover, adolescents until the age of 21 years in Germany are often still either dependent on foster/adoptive parents or living in institutionalized care with some form of supervision. Hence, the entire research consortium implemented a cutoff of 21 years. Further inclusion criteria are sufficient writing and reading skills in German based on self-report and access to the internet via a desktop computer, laptop, tablet, or other mobile device.

#### Exclusion Criteria

Participants are excluded from the RCT when (1) acute child endangerment is suspected; (2) they receive other psychosocial approaches aimed at victimization or revictimization; (3) severe depression symptoms are present as assessed via the Patient Health Questionnaire-9 for Adolescents (PHQ-9) [32] (PHQ-9 >15 at baseline); and (4) suicide ideation (indicated by a score of >1 on the suicidal thought item of the PHQ-9 at baseline) is present. If participants score above 1 on the suicide item, a further suicide screening will be conducted via telephone in accordance with our safety protocol. If participants do not provide a telephone number, either an email or letter by post will be sent to make contact with the participants or their legal guardians.

### Intervention

#### Experimental Condition

The internet-based prevention program EMPOWER YOUTH for adolescents and young adults has been developed to meet the needs of youth in care and address several risk factors of victimization or revictimization. Youth in care who participate are taught theory and skills to prevent victimization in themselves and others. EMPOWER YOUTH promotes knowledge of risk situations and consequences, and strategies that help defend youth from victimization. After finishing the program, the participants receive a certificate stating they are EMPOWER YOUTH coaches. The aspect of becoming a coach and participating to help others is emphasized during recruitment and during the program participation. This program format was chosen because the results of focus groups indicated that youth in care feel stigmatized receiving a program aimed specifically at them as potential “victims.” This change of perspective, that is, being a coach to help others rather than being victimized, prevents youth in care from perceiving themselves as potential victims due to the program, which would directly counteract the aim of the project. The intervention includes various multimedia features (videos, fictional audio recordings, and writing exercises). Participants will be provided with a personalized password-protected interface. Altogether, there will be 6 modules, with an approximately 45-minute workload. Mentors/psychologists will provide individual written feedback within 2 working days, along with instructions for the next module. The mentor contact should enhance compliance with the program [33]. Participants will receive up to three reminders to complete a module.

Based on the existing literature and the evaluation of 3 focus groups with adolescents and young adults in foster care, the 6 program modules focus on the following categories of psychological risk factors: (1) emotion regulation, (2) self-appraisal, (3) risk recognition, (4) offline risk recognition problems and coping with victimization or revictimization, and (5) online risk recognition problems and coping with victimization or revictimization. In all the modules, interpersonal relatedness and prosocial behaviors are indirectly addressed via case descriptions and the accompanying exercises. The case descriptions are partly derived from situations described by youth in care in the initial focus groups.

In the first module “What’s up?”, participants receive knowledge about emotions in a video (naming emotions; regulation of emotions; and the connection between emotions, thoughts, and actions/behaviors). They practice identifying emotions and thoughts in 2 case descriptions (presented as audio recordings) and are asked to reflect on potential actions that could be taken in the described situations. The module also entails an exercise on breathing, an exercise on formulating positive thoughts, and a writing exercise in which the participants describe a difficult biographical situation and identify their emotions, thoughts, and actions in that situation.

The second module “I am okay, you are okay, we are okay,” teaches participants about personal rights, diversity, and self-worth in a video. This is followed by a multiple-choice exercise, in which they identify the personal rights they have recently used, and an exercise on self-care activities. The participants practice identifying personal rights and personal worth in 2 case descriptions, and are asked to reflect on actions they could take as coaches to ensure these personal rights and why these actions should be taken (eg, diversity and general worth). In another exercise, the participants are asked to reflect on obstacles they have overcome, and on their uniqueness and identity as a person. This is followed by a final writing exercise in which participants are asked to write a letter to themselves naming characteristics and past behaviors they are proud of.

In the third module “Stop!”, the participants learn about recognizing (social) risk situations based on different warning signals. They are asked to reflect on the danger presented in 3 different case descriptions. Moreover, several different risk behaviors are listed, and they are asked to rate how likely it is that they would engage in these behaviors. In the final writing exercise, participants are asked to describe a risk situation they found themselves in and to identify the different warning signals they recognized or maybe missed.

After learning how to recognize risk situations, the fourth module “Your limit” deals with effectively setting boundaries in offline risk situations. Four case descriptions are used in this module. The participants practice to differentiate between behavior and emotion, and to identify if these are passive, aggressive, or assertive. In a writing exercise, they are asked to reflect on an offline risk situation they found themselves in; if and how they set boundaries; and if this was passive, aggressive, or assertive. Moreover, they are asked for advice for other adolescents and young adults who could find themselves in similar situations.

The fifth module “Like and share?” deals with setting boundaries in online risk situations. The participants learn about their rights in online situations and how to legally collect evidence and set boundaries. Four case descriptions are used. In a writing exercise, participants are asked to reflect on the module and describe their own personal take-home message that they will use to support others in the future.

In the sixth and final module “You’re the expert,” participants are invited to fill out 2 interactive quizzes. The first quiz consists of claims and asks if these claims are true or false (knowledge). The second quiz consists of 5 case descriptions, and participants are asked what advice, as EMPOWER YOUTH coaches, they have in these situations. Another relaxation method is presented to the participants. The final module ends with a writing exercise in which the participants are asked to reflect on all the modules: what do they take away from the program and what was most important to them?

EMPOWER YOUTH can be accessed online [34]. The development of a website, compatible with a wide variety of electronical devices, such as desktop computers, laptops, and mobile phones with different operating systems, increases program participation. The use of a mobile app would have created an unwanted selection bias.

#### Control Condition

For ethical reasons, a waiting-list control group has been chosen. A placebo comparator has not been chosen, because there is no evaluated similar intervention for this condition in this specific population.

#### Concomitant Treatment

Additional treatments can be administered on entry into the trial or at any time during the study. These will be documented at the follow-up assessment as concomitant treatments on the case report form of the participant. Concomitant treatments are allowed in the intervention group as well as the control group if they do not represent psychosocial approaches aimed at victimization or revictimization. Examples of concomitant treatments include individual psychotherapy, psychiatric consultations, and activities that serve to strengthen social-emotional, cognitive, or physical competencies.

#### Allocation

Randomization will take place after the baseline assessment. The randomization procedure will be conducted by employees at the University Hospital Aachen (part of the EMPOWERYOU consortium), which will not be involved in this trial otherwise. Hereby, a blind randomization process is guaranteed. Participants will be informed of their allocation status via email after baseline data collection is completed to assure that they are blind to allocation during the initial assessment. Participants will be randomized into the following 2 groups: intervention group and waiting-list control group. The allocation ratio between the 2 arms of the study is 1:1. After 18 weeks, a follow-up assessment will take place. Each assessment lasts approximately 45 minutes and is conducted online. For the online assessments, the participants will receive an invitation via email with an individual identification code (subject number and randomization number). This allows for data collection without any direct identifiers.

### Outcome Measures

The primary endpoint is assessed via a composite score of the Bullying Screener [35] and the Juvenile Victimization Questionnaire (JVQ) [36]. The Bullying Screener is a 6-item screening tool that assesses bullying as a victim and an offender. After the respective definition of bullying type, children are asked how often these things have happened to them or how often they have done this to others in the last 3 months. Children then respond on a 4-point scale from never to a lot (at least once a week) [35]. The JVQ is a validated screening questionnaire including 34 offenses against youth and covers the following 5 areas of concern: (1) conventional crime, (2) child maltreatment, (3) peer and sibling victimization, (4) sexual victimization, and (5) witnessing and indirect victimization. It encompasses follow-up questions that also assess the frequency and perpetrators of the victimization events. Children are asked whether they were exposed to the respective event in the past 3 months (time period adaptation for this study) and respond with yes (1) or no (0), leading to a total score, with a higher score indicating greater victimization exposure [36].

The secondary endpoints are risk-taking behavior, recognition of problematic/dangerous situations, aggressive tendencies, empathy, prosocial behaviors, depressiveness, posttraumatic stress symptoms, and loneliness. The Adolescent Risk-taking Questionnaire [37] will be used to measure risk-taking behaviors and judgements. It consists of 2 sections. The first section of the questionnaire measures adolescents’ judgements of riskiness for 22 behaviors, and the second part measures adolescents’ frequency in engaging in these behaviors. Judgement of riskiness is made on a 5-point Likert scale, ranging from 0 (not at all risky) to 4 (extremely risky). Participation in risky behavior was also rated on a 5-point Likert scale, ranging from 0 (never done) to 4 (done very often). The total risk judgement score is obtained by adding all the items; a high score indicates a strong judgement of riskiness for the behaviors listed in the questionnaire. The total risk behavior score is obtained by adding the frequency rating of all the items; a high score indicates a high level of participation in risky activity. The items can be divided into the following 4 major factors: thrill-seeking risks, rebellious risks, reckless risks, and antisocial risks. The Cronbach α coefficient for the risk judgement scale is .97 (range .86±.96) and for the behavior scale is .99 (range .87±.96). A good test-retest reliability was found, with 1-week test-retest reliability for risk judgement being 0.79 and for risk behavior being 0.78 [37].

The following constructs will be assessed via 3 of the 4 subscales of the German Questionnaire for determining empathy, prosocial behavior, and aggression (FEPAA) [38]. There is an alternative version (versions A and B), and different versions will be used at the 2 assessment points. Cronbach α varies between .61 (prosocial behavior scale) and .79 (aggressive tendencies scale) for version A, and between .57 (prosocial behavior scale) and .77 (aggressive tendencies scale) for version B. Reliability is 0.75 for version A and 0.66 for version B. Overall, 40 items of the original 55 will be assessed.

Depressiveness will be assessed with the PHQ-9 [32]. The PHQ-9 is used to screen for the presence and severity of depression and takes less than 3 minutes to complete. The PHQ-9 achieved a Cronbach α of .89 among 3000 primary care patients. The test-retest reliability was assessed by the correlation between PHQ-9 scores obtained from in-person and phone interviews with the same patients. The correlation value obtained was 0.84 [32].

Loneliness will be assessed via the Loneliness Scale-SOEP [39], which consists of 3 items with a 5-point rating scale. The α coefficient of reliability obtained was .72 [39].

To assess the presence of any posttraumatic stress symptoms, the 8-item version of the Child Revised Impact of Events Scale [40] will be used. It consists of items with a 4-point rating scale and takes between 5 and 10 minutes to complete.

### Statistical Analysis

An intention-to-treat design will be used. Missing data due to study dropout will be handled using multiple imputation. E-mental health programs for children, adolescents, and young adults with the highest rate of completion were those with therapist support, with dropout rates ranging from 13% to 17% [33]. In our trial, we anticipate a dropout rate of 30% at follow-up assessment, using a more conservative attrition rate and following the results of our pilot feasibility study. At the end of the trial, a dropout analysis will be conducted.

#### Primary Endpoint

The primary endpoint (number of victimizations 18 weeks after randomization) will be analyzed by analysis of covariance (ANCOVA) with baseline individual number of victimization scores, self-reported depression, severity of victimization (eg, sexual abuse), and PTSD as covariates and intervention as a factor. A significance level of 5% will be chosen. No interim analysis is planned. Conservative missing value imputation strategies will be performed if necessary.

#### Secondary Endpoints

Quasimetric scores of self-reported questionnaires will be analyzed in an exploratory manner and will also be evaluated by ANCOVA, if suitable, regarding the scale level and type of distribution. Otherwise, they will be transformed to fulfill the presuppositions of the method or will be analyzed by means of nonparametric methods. For categorical secondary endpoints, absolute and relative frequencies will be presented and tested by the chi-square test or Fisher exact test, as appropriate. In case significant treatment differences are observed between both arms, potential predictors of beneficial treatment outcomes (eg, sociodemographic or individual ones) will be identified using multivariable analyses (eg, logistic regression models). To investigate the predictors of beneficial protocol adherence and dropout, logistic regression models will be applied. For all primary and secondary endpoints, quartiles and, if suitable, means and standard deviations will be reported for descriptive purposes. Effect sizes will be estimated and presented with 95% CIs. Predictors of treatment outcome will be identified using multivariable analyses. For secondary analyses, neither adjustment for multiple testing nor imputation of missing values is planned. Quartiles and, if suitable, means and standard deviations will be reported for descriptive purposes. 

#### Power

The expected outcome requires a sample size of 156 subjects (α=.05, *t* test, 1-sided) to achieve a power of 80%. These assumptions are based on the effects (*d*=0.40) of previously published studies [41] on follow-up change in the revictimization composite score. Assuming a 30% dropout at follow-up, we require 225 participants to be allocated to the trial. In the middle of the trial, the dropout rate will be assessed in an interim evaluation, and in case of a higher dropout rate than expected, the number of participants allocated to the trial will be increased accordingly.

### Ethical Considerations

The study has been approved by the Ethics Committee of the Medical School Berlin (MSB-2021/55). Informed consent in the study will be obtained from the participants before baseline assessment via a digital double opt-in method. For participants under the age of 16 years, informed consent from a legal guardian will be obtained before baseline assessment. Participants have the possibility to leave the trial without any disadvantage at any time. The trial will be conducted in accordance with the Guidelines for Good Clinical Practice (ICH-GCP), the Declaration of Helsinki (latest version), and international and local laws. The study has been registered in the German Clinical Trials Register (DRKS00024749). Throughout the trial, participants will be identified solely by means of an individual identification code (subject number and randomization number). Electronic case report forms will be stored in accordance with local data protection laws and will be handled with the strictest confidence. The appropriate regulations of local data legislation (ie, European General Data Protection Regulation [42]) will be fulfilled in its entirety.

### Data Safety and Monitoring

#### Data Safety

Participants will be deidentified, including the removal of direct identifiers (eg, names and addresses) and indirect identifiers (eg, occupation). Nonelectronic data will be stored in a locked filing cabinet at the university. These data will be kept for 10 years. Electronic data will be kept on 2 password-protected servers only accessible by approved study staff members.

#### Data Monitoring and Auditing

An independent Data Safety Monitoring Board (DSMB) has been established. The DSMB will provide additional oversight on data safety, ethical procedures, and best clinical practice. This DSMB is independent of all investigators and the funding agency, and no member of the DSMB has direct involvement in the conduct of the study. The DSMB is composed of 3 researchers familiar with the area of the study. The type of information monitored will include recruitment, number of dropouts, and all adverse events, including study withdrawals. The DSMB will receive recruitment and retention updates on a regular basis. Before starting with the trial, a data safety concept was developed and approved by the DSMB. No external auditing is planned.

#### Stopping Rules

A participant may withdraw from the study at any time, at his or her own request. Upon request, all collected data (ie, from the assessments and responses in the online program) will be deleted. The responsible investigator has the right to discontinue the intervention for a participant who experiences one or more of the following incidents: (1) adverse events or serious adverse events, particularly acute child endangerment or suicidal tendency and (2) an unacceptable benefit/risk ratio. In case a family member reports aversive experiences during the trial, we will follow recommendations for the ethical treatment of participants and provide referrals for services. In cases where acute child endangerment is detected, we will report this to the appropriate services, and based on the recommendations from the DSMB, we will halt study participation. However, youth in care will still be able to complete the intervention if desired. The responsible investigator has the right to discontinue the whole trial, if information that affects the benefit/risk ratio of the trial emerges or if there are repeated serious adverse events associated with the trial. In this case, the decision of stopping the trial will be communicated to the DSMB and discussed with all principal investigators of the EMPOWERYOU consortium.

## Results

Ethical approval was granted by the Ethics Committee of the Medical School Berlin in March 2021 (MSB-2021/55). Recruitment started in September 2021 and is planned until November 2022. The results are expected to be published in January 2023.

## Discussion

Given the increased likelihood of future victimization experiences among youth in care, there is a strong need for a low-threshold intervention specifically for this high-risk age group. So far, there are no existing nation-wide mental health programs exclusively for youth in care in Germany. If the efficacy of the prevention program EMPOWER YOUTH is identified in the RCT, a widespread implementation and dissemination process is planned. Stakeholders have been involved in the developmental phase and will consequently be informed of the RCT results via nontechnical briefs, symposia, conference presentations, and publications. Moreover, as part of the integrated knowledge translation, social media channels will post regular updates on the project. Providing knowledge to child welfare workers, social workers, medical doctors, and psychotherapists about the issue of victimization will support the implementation of EMPOWER YOUTH as a potential prevention program. This will ultimately improve the well-being of youth in care and prevent the need for more intensive and costly service utilization for youth in care. The future use of EMPOWER YOUTH could be seen under the Prevention Act, an initiative to promote health. Health insurance could support the continuation of the prevention program after the end of funding.

## Abbreviations

ANCOVA: analysis of covariance
DSMB: Data Safety Monitoring Board
JVQ: Juvenile Victimization Questionnaire
PHQ-9: Patient Health Questionnaire-9 for Adolescents
PTSD: posttraumatic stress disorder
RCT: randomized controlled trial
